# Presence and abundance of malaria vector species in Miami-Dade County, Florida

**DOI:** 10.1186/s12936-024-04847-9

**Published:** 2024-01-18

**Authors:** André B. B. Wilke, Chalmers Vasquez, Johana Medina, Isik Unlu, John C. Beier, Marco Ajelli

**Affiliations:** 1 Laboratory for Computational Epidemiology and Public Health, Department of Epidemiology and Biostatistics, Indiana University School of Public Health, Bloomington, IN USA; 2 Miami-Dade County Mosquito Control Division, Miami, FL USA; 3 Department of Public Health Sciences, Miller School of Medicine, University of Miami, Miami, FL USA

**Keywords:** *Anopheles crucians*, *Anopheles quadrimaculatus*, Epidemiology, *Plasmodium*, Re-emerging arthropod borne pathogen

## Abstract

**Background:**

Malaria outbreaks have sporadically occurred in the United States, with *Anopheles quadrimaculatus* serving as the primary vector in the eastern region. *Anopheles crucians*, while considered a competent vector, has not been directly implicated in human transmission. Considering the locally acquired *Plasmodium vivax* cases in Sarasota County, Florida (7 confirmed cases), Cameron County, Texas (one confirmed case), and Maryland (one confirmed case) in the summer of 2023. The hypothesis of this study is that major cities in the United States harbour sufficient natural populations of *Anopheles* species vectors of malaria, that overlap with human populations that could support local transmission to humans. The objective of this study is to profile the most abundant *Anopheles* vector species in Miami-Dade County, Florida—*An. crucians* and *An. quadrimaculatus*.

**Methods:**

This study was based on high-resolution mosquito surveillance data from 2020 to 2022 in Miami-Dade County, Florida. Variations on the relative abundance of *An. crucians* and *An. quadrimaculatus* was assessed by dividing the total number of mosquitoes collected by each individual trap in 2022 by the number of mosquitoes collected by the same trap in 2020. In order to identify influential traps, the linear distance in meters between all traps in the surveillance system from 2020 to 2022 was calculated and used to create a 4 km buffer radius around each trap in the surveillance system.

**Results:**

A total of 36,589 *An. crucians* and 9943 *An. quadrimaculatus* were collected during this study by the surveillance system, consisting of 322 CO_2_-based traps. The findings reveal a highly heterogeneous spatiotemporal distribution of *An. crucians* and *An. quadrimaculatus* in Miami-Dade County, highlighting the presence of highly conducive environments in transition zones between natural/rural and urban areas. *Anopheles quadrimaculatus*, and to a lesser extent *An. crucians*, pose a considerable risk of malaria transmission during an outbreak, given their high abundance and proximity to humans.

**Conclusions:**

Understanding the factors driving the proliferation, population dynamics, and spatial distribution of *Anopheles* vector species is vital for implementing effective mosquito control and reducing the risk of malaria outbreaks in the United States.

## Background

Malaria is an infectious disease caused by *Plasmodium* species parasites [1]. *Plasmodium* parasites are primarily transmitted to humans through the bites of infected female *Anopheles* mosquitoes (Diptera: Culicidae). Malaria continues to pose a significant public health challenge, particularly in tropical and subtropical regions of Africa, Asia, and South America [2–6]. Africa is the most affected region concentrating most cases and deaths [7]. Although effective drugs are available [8, 9], the gold standard for preventing malaria transmission remains the control of *Anopheles* mosquitoes [10, 11].

Malaria used to be endemic in the United States (USA) until the 1950s. However, sporadic malaria outbreaks in the USA have been reported since then [12–15]. *Anopheles quadrimaculatus* and *Anopheles freeborni* are the primary vectors of malaria in the USA [13, 16]. *Anopheles crucians* is considered a competent vector, but has not been directly implicated in malaria transmission to humans [17]. *Anopheles freeborni* is commonly found in the western part of the USA, while *An. quadrimaculatus* and *An. crucians* are more prevalent in the eastern regions [18–21]. The anthropophilic behaviour of *An. quadrimaculatus* and, to a lesser extent, *An. crucians* make these species significant threats for malaria transmission in the USA [22, 23].

*Anopheles quadrimaculatus* and *An. crucians* were abundant in Palm Beach County, Florida, and implicated as probable vectors during the 2003 malaria outbreak [14], and all seven cases were attributed to *P. vivax*. Construction workers and unsheltered homeless individuals were the most affected, underscoring the role of social and economic disparities in the risk of mosquito-borne disease transmission in the USA [14]. In 2023, after 20 years without reported locally acquired malaria infections in the continental USA [24], the Centers for Disease Control and Prevention (CDC) responded to locally acquired *P. vivax* malaria cases. A total of 9 cases were reported, seven in Sarasota County, Florida, one in Cameron County, Texas, and one in Maryland [15, 25]. Three of the seven cases in Sarasota County occurred in people experiencing homelessness [26].

Given the recent history of malaria outbreaks in large-populated areas of the USA, the hypothesis of this study is that other areas harbour sufficient natural populations of *Anopheles* species vectors of malaria that overlap with human populations and that could support local transmission to humans. Miami-Dade County, Florida, is one of the most important gateways into the USA. Miami-Dade County is not only one of the most important tourist destinations, receiving an average of over 120 million visitors every year, but is also a pivotal operational hub for the cruise ship industry, serving as the main port for cruise ships sailing to the Caribbean and Gulf of Mexico [27]. Miami-Dade County is also a hub for cargo ships that routinely transport goods between Miami-Dade County and Caribbean countries, including Haiti, Dominican Republic, and Cuba, increasing the risk of pathogen importation into the USA [28]. The 2023 malaria outbreak in Sarasota County—approximately 300 km northwest of Miami-Dade County—highlights the need to improve surveillance and outbreak preparedness and response to mitigate the increasing threat of malaria in Miami-Dade County and other similar potentially high-risk areas of the USA.

To effectively control mosquito populations and mitigate the risk of disease transmission, the strategic use of traps to identify areas with higher mosquito abundance and providing valuable data for informed decision-making is essential to focus resources on locations where mosquito populations pose the most significant threat to public health. The objective of this investigation is to derive a spatial and temporal profile of the malaria vector species *An. crucians* and *An. quadrimaculatus* in Miami-Dade County, Florida, and to identify influential traps to serve as early warning systems. Recognizing hotspot areas and influential traps enables strategic mosquito control operations, focusing efforts on locations favourable for mosquito proliferation and identifying local-level drivers supporting their population growth. Results from this study will guide and target finite resources for mosquito control strategies and enhance preparedness and response measures for potential malaria outbreaks.

## Methods

### Mosquito surveillance system

**Fig. 1:**
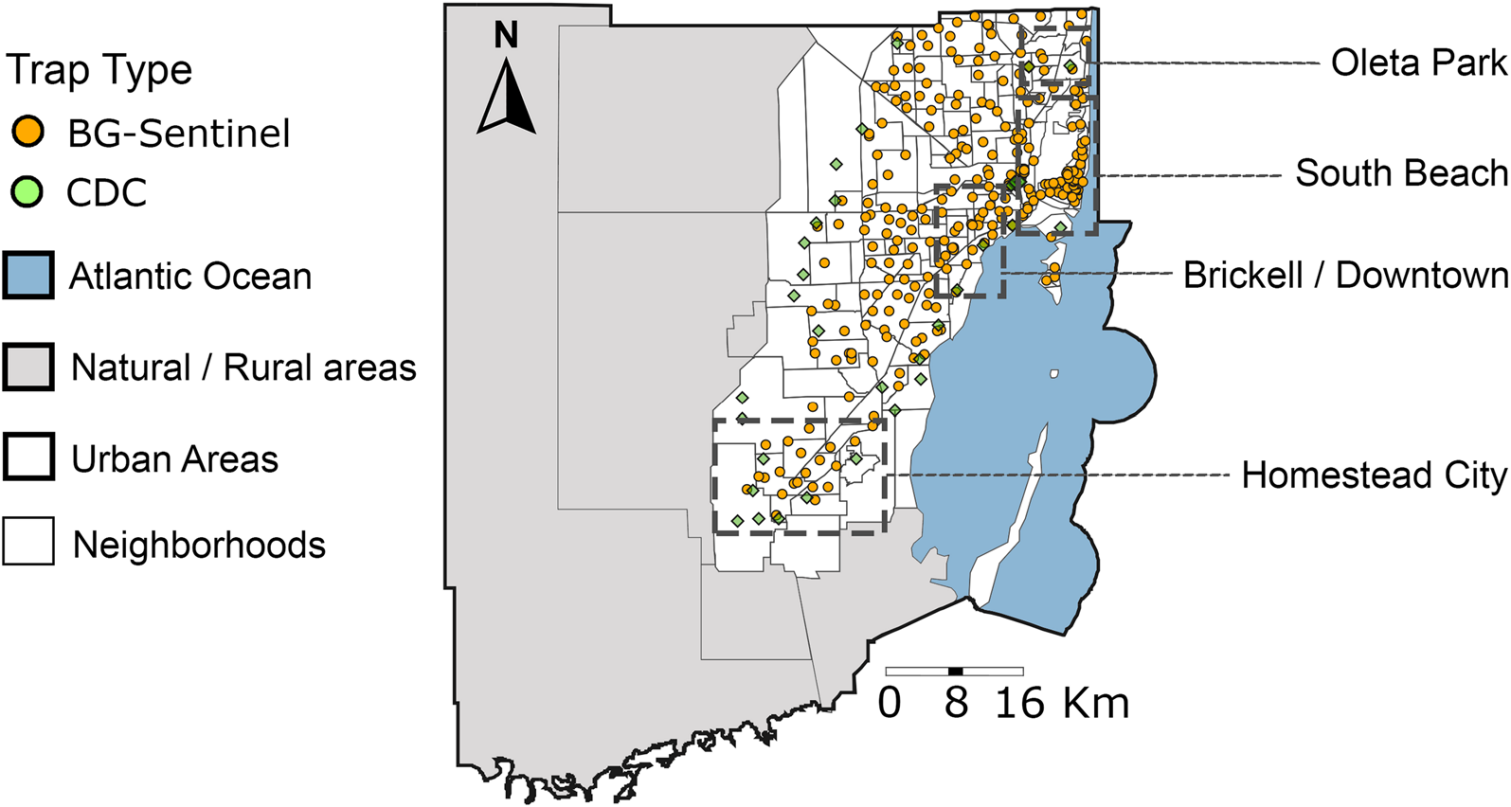
Mosquito surveillance system. Spatial distribution of traps in Miami-Dade mosquito control surveillance system as of 2022. BG-Sentinel traps are represented by orange circles; CDC traps are represented by green squares

The Miami-Dade County Mosquito Control surveillance grid currently consists of 322 traps, including 283 BG-Sentinel traps (Biogents AG, Regensburg, Germany) and 39 CDC traps (Fig. 1). In 2020, the surveillance system was comprised of 211 BG-Sentinel and 36 CDC traps. In 2021, 72 additional BG-Sentinel and 3 CDC traps were added to the surveillance system. In 2022, no traps were added. During the study period, January 2020 to December 2022, each trap was deployed every week for 24 h. All BG-Sentinel and CDC traps (without a light source) were baited with CO_2_ using a container filled with 1 kg of dry ice pellets [29]. All 322 traps were used in this study. All collected mosquitoes were transported to the Miami-Dade County Mosquito Control Laboratory and morphologically identified using taxonomic keys [30]. Although both BG-Sentinel and CDC traps are designed to attract and collect host-seeking female mosquitoes, male mosquitoes were occasionally present in small numbers in collections; however, male mosquitoes were not included in the analysis.

*Anopheles crucians*, *Anopheles bradleyi*, and *Anopheles georgianus* are considered a species complex (Crucians Complex) and are indistinguishable as adults. Since only adult mosquitoes were collected in this study, they are referred to as *An. crucians*. The majority of the mosquitoes from the Crucians complex collected in this study were not collected in association with salt marshes (often associated with *An. bradleyi*) but further inland associated with freshwater.

### Ratio analysis

To assess variations in the relative abundance of *An. crucians* and *An. quadrimaculatus*, the ratio was calculated by dividing the total number of mosquitoes collected by each individual trap in 2022 by the number of mosquitoes collected by the same trap in 2020. Traps that were not part of the surveillance system in 2020 were excluded from the ratio analysis.

### Influential traps analysis

To identify conducive areas for the proliferation of mosquitoes and identify influential traps for the early detection of mosquito abundance increases, the same approach used in Wilke et al. [31] was followed. Briefly, the linear distance in meters between all traps in the surveillance system from 2020 to 2022 was calculated and used to create a 4 km buffer radius around each trap in the surveillance system. A buffer size of 4 km was established because it enclosed an optimum number of traps in each buffer (more than 15 traps per buffer) to enable a robust statistical analysis and still maintain sufficient spatial resolution for informing local control operations. Traps with no data were removed from the analysis (146 traps did not collect *An. crucians* and 128 traps did not collect *An. quadrimaculatus*). To calculate mean buffer values, outliers were excluded within each buffer by eliminating observations lying outside the expected range of mean variability, i.e. excluding values above or below ${median}\pm 1.58\times \frac{IQR}{\sqrt{n}}$, where *IQR* is interquartile range and *n* is the number of observations in the buffer. This analysis was conducted in R version 4.2.2.

## Results

In 2020, a total of 10,638 *An. crucians* specimens were collected, 20,844 in 2021, and 5107 in 2022, summing up to 36,589 specimens collected over the three years of this study. A total of 2189 specimens of *An. quadrimaculatus* were collected in 2020, 4151 in 2021, and 3603 in 2022, for a total of 9943 specimens collected during the study period.

**Fig. 2:**
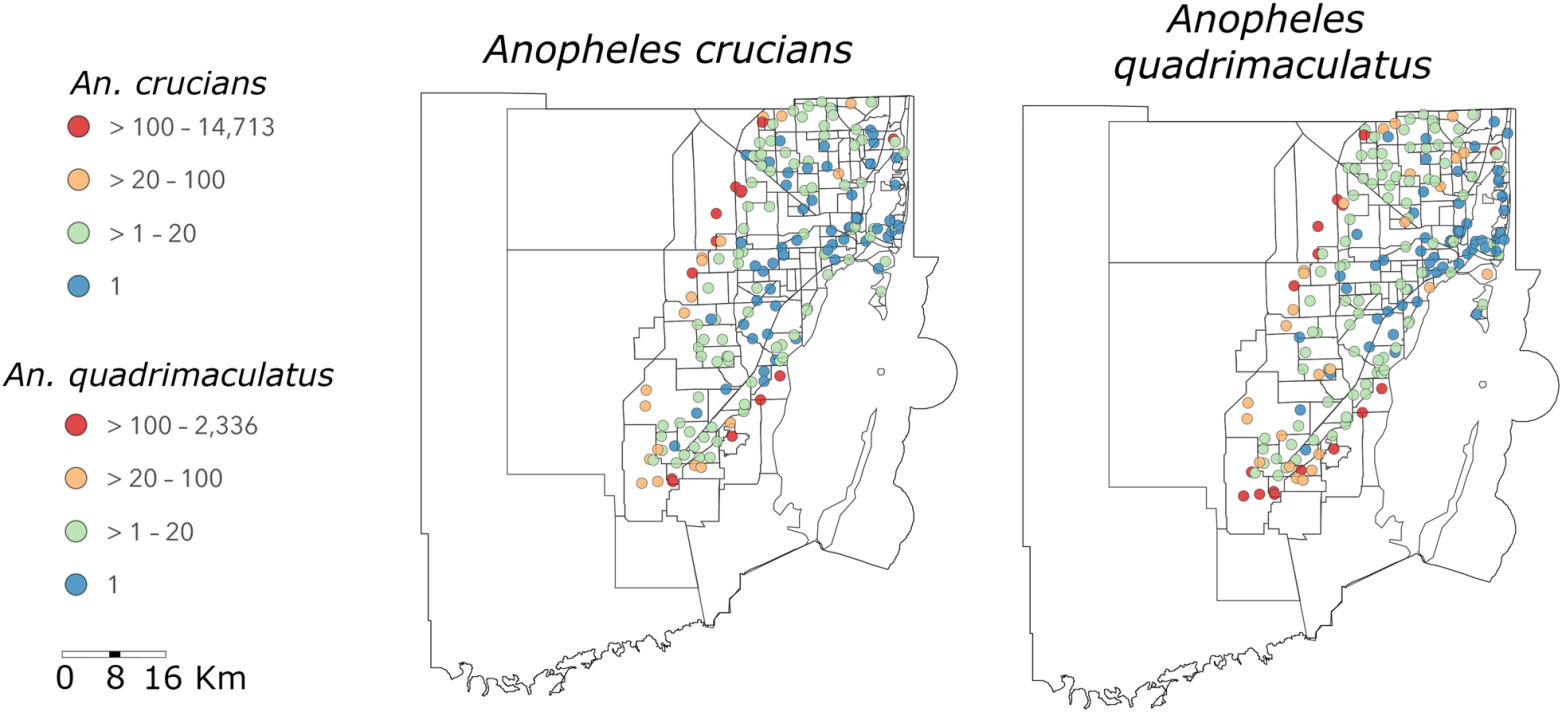
Spatial distribution of total *An. crucians* and *An. quadrimaculatus* collected over the 3 years study period. Total numbers of *An. crucians* and *An. quadrimaculatus* collected in 2020, 2021, and 2022 in Miami-Dade, Florida, classified into four numerical ranges. The groupings in these ranges demonstrate that a small number of significant trap locations collected the majority of both species

Both *An. crucians* and *An. quadrimaculatus* were abundant in transition zones between natural and urban areas and reached high abundances in specific locations in the northwestern and western parts of the county. These specific locations accounted for 10% of the traps collecting > 90% of mosquitoes, with individual traps collecting up to 14,713 *An. crucians* and 2336 *An. quadrimaculatus* over the 3-year period. *Anopheles quadrimaculatus* was also abundant in the southern part of Miami-Dade County, with several traps collecting a high number of specimens in recently urbanized areas. Both species were also abundant in Oleta Park in the northeast region of the county (Fig. 2).

**Fig. 3:**
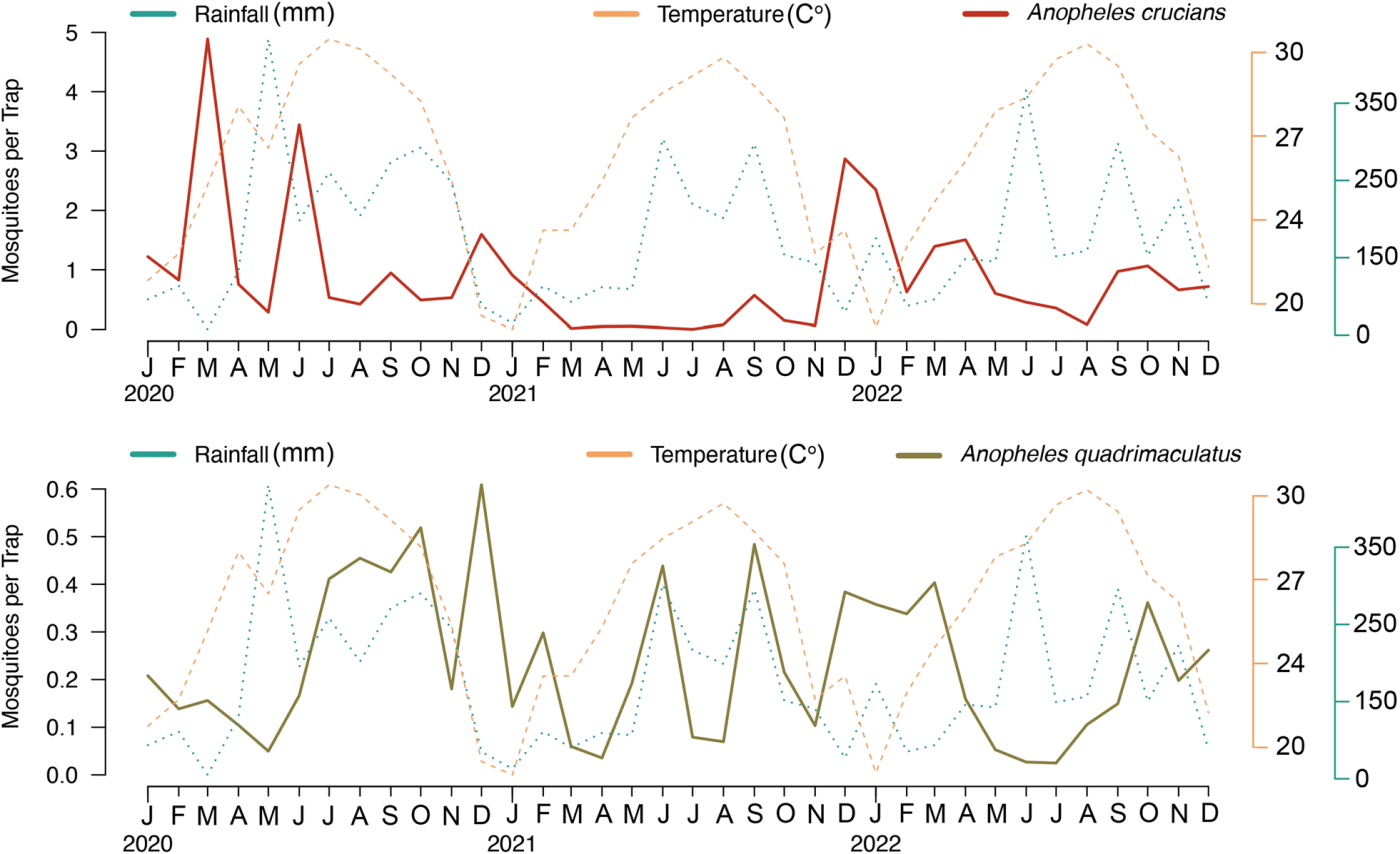
Average number of collected *An. crucians* and *An. quadrimaculatus* per month by the mosquito surveillance system from January 2020 to December 2022

The weekly collection of mosquitoes showed that the population dynamics of *An. crucians* and *An. quadrimaculatus* was highly heterogeneous, showing no clear seasonal trend (Fig. 3). Among *An. crucians*, the numerically highest average number of specimens per trap was observed in March 2020, with an average of 4.88 mosquitoes collected per trap per month, followed by 3.44 in June 2020, and 2.86 in December 2021. Conversely, the lowest averages were recorded in July 2021, with an average number of 0.002 *An. crucians* collected per trap. For *An. quadrimaculatus*, the highest average number of specimens per month occurred in December 2020, with an average of 0.6 mosquitoes collected per month, followed by 0.51 in October 2020, and 0.48 in September 2021. The lowest averages were observed in July 2022, with an average number of 0.02 *An. quadrimaculatus* collected per trap. No clear association between the temporal dynamics of the two species with temperature and rainfall was observed (Fig. 3).

**Fig. 4:**
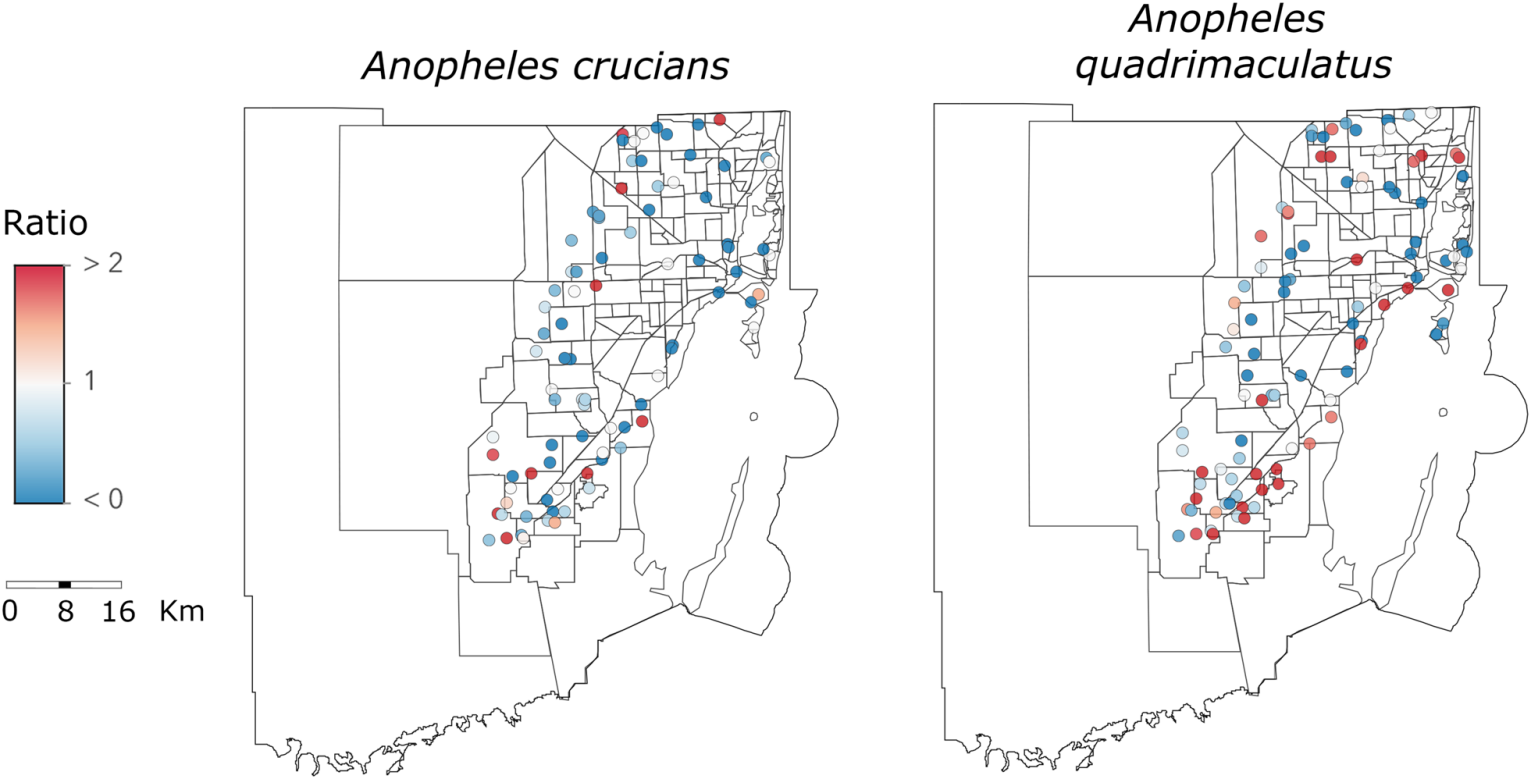
Relative change in mosquito relative abundance. Ratio of *An. crucians* and *An. quadrimaculatus* collected by traps in 2022 compared to 2020

The ratio analysis revealed substantial fluctuations in the abundance of *An. crucians* and *An. quadrimaculatus* in specific areas of Miami-Dade County. *Anopheles crucians* increased in abundance in 14 traps in the southern and western parts of the county; however, 28 traps showed a decrease in the number of collected specimens (Fig. 4). *Anopheles quadrimaculatus* increased in abundance in 34 traps, including in 12 traps located in the southern part of the county (i.e., Homestead) that has recently undergone intense urbanization [32], and is one of the last stops for tourists going to the Florida Keys. On the other hand, the number of *An. quadrimaculatus* collected in the western part of the county decreased over time.

**Fig. 5:**
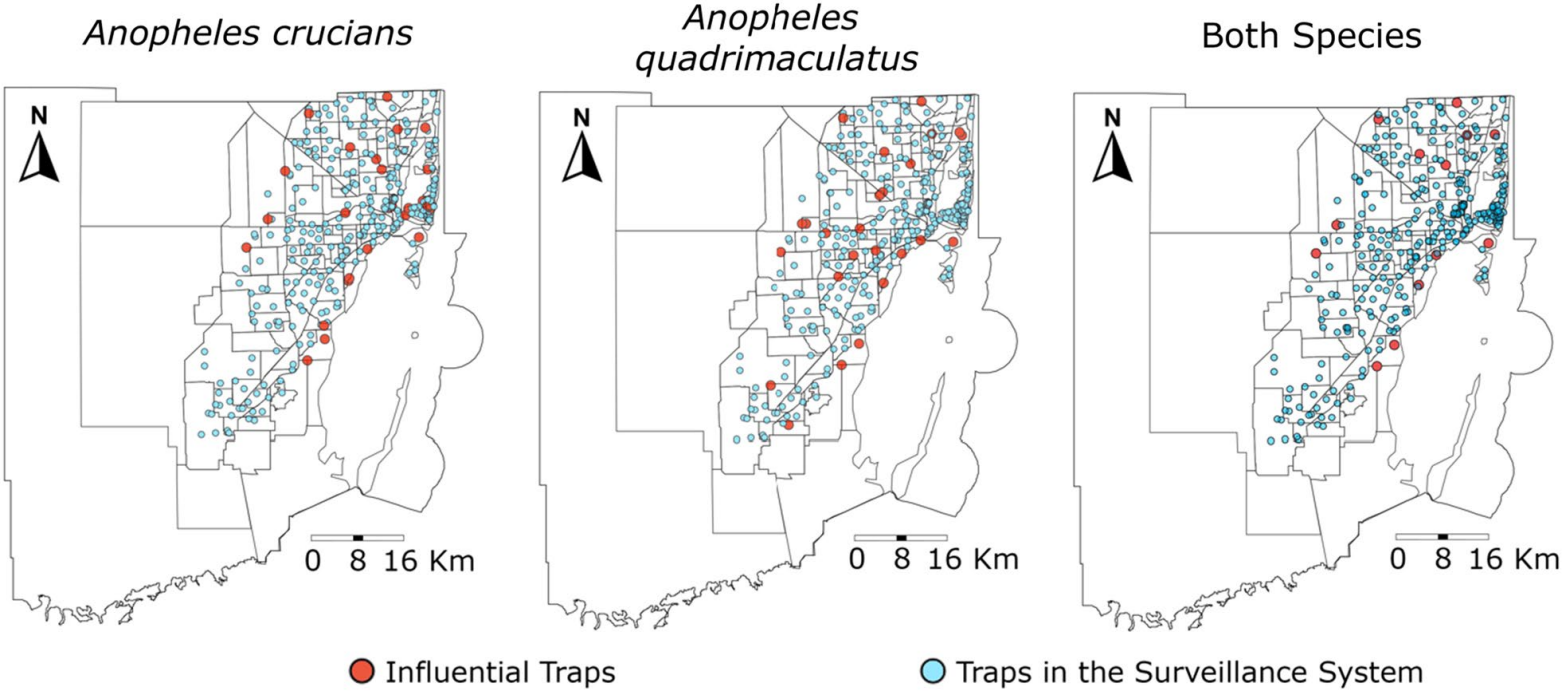
Spatial distribution of influential traps. Traps that collected a higher (red) number of female *An. crucians* and *An. quadrimaculatus* mosquitoes relative to the traps in their respective 4 km buffer radius are identified as influential traps. Represented in blue are the remaining traps of the surveillance system

The identification of areas conducive to mosquito proliferation and influential traps (defined as traps yielding an average number of mosquitoes at the 97.5th percentile of the cluster) to serve as early warning systems are vital in detecting increases in mosquito vector species abundance. Recognizing hotspot areas and influential traps enables strategic mosquito control operations, focusing efforts on locations favorable for mosquito proliferation and identifying local-level drivers supporting their population growth. Of the 322 traps in the surveillance system, 176 collected *An. crucians*. From those, 85 collected *An. crucians* above their buffer average and 36 traps were considered influential traps (Fig. 5). Similarly, of the 322 traps in the surveillance system, 194 traps collected *An. quadrimaculatus*. From those, 86 collected *An. quadrimaculatus* above their buffer average and 32 traps were considered influential traps. Moreover, 13 traps were identified as influential traps for both *An. crucians* and *An. quadrimaculatus* (Fig. 5).

## Discussion

The risk of malaria transmission in the USA depends on imported human cases and the presence of mosquito vector species competent for the malaria parasites. The USA reports approximately 2000 imported cases of malaria every year [24], and many regions have established *Anopheles* species mosquito vector populations able to transmit *Plasmodium* parasites, leading to sporadic local malaria transmission [12, 14, 15, 33, 34]. The results show that malaria vectors *An. crucians* and *An. quadrimaculatus* in Miami-Dade County, Florida, are abundant and their spatial distribution highly heterogeneous. Both species were highly abundant in transition zones between natural/rural and urban areas but were not found in great numbers in more urbanized areas within the county. This spatial pattern likely results from *An. crucians* and *An. quadrimaculatus* inherent biological and physiological requirements, which are not adequately met in highly urbanized environments. However, urban areas near natural and rural areas have been shown to be conducive to the proliferation of these species.

The spatial analysis showed that *An. crucians* and *An. quadrimaculatus* are abundant in well-defined areas. Notably, 10% of the traps collected > 90% of all mosquitoes, with individual traps collecting up to 14,713 *An. crucians* and 2336 *An. quadrimaculatus* throughout the study period. In contrast, 90% of the traps collected substantially fewer mosquitoes, with counts ranging below 18 *An. crucians* and 29 *An. quadrimaculatus*.

The comparison between the number of mosquitoes collected by specific traps showed that 28 traps had a decreasing trend in *An. crucians* counts over time and 14 traps displayed a substantial increase. *An. quadrimaculatus* counts over time decreased in 26 traps and 34 traps displayed a substantial increase. For *An. crucians*, the number of collected mosquitoes increased in specific traps situated in well-established transition zones between natural/rural and low-population urban regions. Conversely, *An. quadrimaculatus* exhibited an increase in abundance in several traps located in the southern part of the county, which has been undergoing intense urbanization processes. For example, the population of Homestead, Florida, increased from 60,512 in 2010 to 80,734 in 2022 (~ 33% increase) [35]. However, despite recent intense urbanization processes in the area, Homestead is still surrounded by rural areas associated with crop cultivation. This increased mosquito abundance is likely attributed to the availability of resources in both natural and rural areas such as farm animals and large bodies of water, conducive to the proliferation of *An. quadrimaculatus*, as well as the additional resources available in the recently urbanized areas such as large artificial bodies of water (e.g., human-made ponds, canals) capable of sustaining immature mosquitoes. These findings indicate a heightened level of human exposure to *Anopheles* vectors in specific regions of Miami-Dade County, with a notable increase in exposure specifically to *An. quadrimaculatus* in the southern part of the county. Future studies could be conducted by overlaying landscape features to identify larval habitat adjacent to important traps to improve larval control.

To effectively control mosquito populations and mitigate the risk of disease transmission, the strategic use of traps that are already in use in the surveillance system and were identified by the analysis of the spatial distribution as influential traps play a vital role. In fact, although it is known that *An. quadrimaculatus* prefers open sunlit fresh water-ground pools with slightly alkaline water and abundant floating an/or emergent vegetation and *An. crucians* prefers acidic water in a low light situation such as a cypress swamp, influential traps could serve as early warning systems. They provide quantitative evidence that help identifying areas with higher mosquito abundance to provide valuable data for informed decision-making. This targeted approach allows for the rapid and efficient allocation of resources, focusing on locations where mosquito populations pose the most significant threat to public health. Furthermore, the inclusion of traps that are highly effective in collecting *Anopheles* species (e.g., CDC Fay-Prince) would also increase preparedness and response in case of a malaria outbreak.

## Conclusion

Effective mosquito control strategies are crucial to enhance preparedness and response for dealing with malaria outbreaks in the USA. The high abundance of *An. quadrimaculatus*, and to a lesser extent *An. crucians*, in proximity to humans in Miami-Dade County, pose an elevated risk of malaria transmission. Gaining a better understanding of the drivers enabling the proliferation of *Anopheles* vector species, along with their population dynamics and spatial distribution, is essential to implement effective mosquito control to mitigate the risk posed by the influx of travellers carrying malaria into the USA.

## Data Availability

The data supporting the findings of this study will be made available upon request.
